# Covalent Immobilization of L-Asparaginase and Optimization of Its Enzyme Reactor for Reducing Acrylamide Formation in a Heated Food Model System

**DOI:** 10.3389/fbioe.2020.584758

**Published:** 2020-10-15

**Authors:** Ran Li, Zehua Zhang, Xiaomei Pei, Xiaole Xia

**Affiliations:** The Key Laboratory of Industrial Biotechnology, School of Biotechnology, Jiangnan University, Wuxi, China

**Keywords:** acrylamide, asparaginase, immobilization, packed bed reactor, kinetic model

## Abstract

Acrylamide is a potent carcinogen and neurotoxin that is mainly formed by the Maillard reaction of asparagine with starch at high temperatures. In this work, a food safety immobilization system for L-asparaginase (L-ASNase) consisting of food-grade agarose (Aga) spheres and N-hydroxysuccinimide esters was developed to decrease the formation of acrylamide in a fluid food model system. L-asparaginase was successfully immobilized with a maximum immobilization efficiency of 68.43%. The immobilized enzymes exhibited superior storage stability and reusability with 93.21 and 72.25% of the initial activity retained after six consecutive cycles and storage for 28 days, indicating its high industrial application potential. Meanwhile, a simplified mathematical model of the enzyme reactor was developed and verified with experiments, which demonstrated its auxiliary role in the design and optimization of reactors. In addition, simulated fluidized food components were continuously catalyzed in the designed packed bed reactor, achieving a reduction rate of nearly 89%.

## Highlights

- Food safety immobilization system of L-asparaginase, which consist of food-grade agarose microsphere and NHS ester.
- The storage stability and reusability is better than a previous report.
- Mathematical modeling directed optimization of packed-bed continuous enzyme reactor, which gives a strong operation performance in the degeneration of acrylamide in a fluidized food system.

## Introduction

L-asparaginase (L-ASNase, EC3.5.1.1) can specifically catalyze L-asparagine conversion to ammonia and L-aspartic acid. Due to its antineoplastic ability, L-asparaginase has been widely studied as a potential treatment for acute lymphoblastic leukemia and lymphosarcoma (Mu et al., 2014; Ulu et al., 2018). In addition, L-asparaginase can inhibit acrylamide formation in a variety of heat-processed starch-rich foods. Acrylamide is a potential human carcinogen with neurotoxic and genotoxic effects (Tyl and Friedman, 2003) and is often detected in diverse starch-rich foods after frying, baking, or grilling (Mottram et al., 2002). Reducing L-asparagine levels in raw materials by adding free L-asparaginase has been proposed as an effective measure to minimize acrylamide in heat-processed foods (Kukurova et al., 2009). For example, supplementing 10 U of asparaginase per gram of flour could effectively reduce acrylamide by 90% during the baking process for bread and biscuits (Anese et al., 2011). However, the reportedly poor instability and recyclability of free L-asparaginase increase the cost of food processing and limit the large-scale usage of these enzymes.

Recently, to increase the stability and recyclability of L-asparaginase, many materials, such as albumin, dextran, chitosan, fructose, inulin, PEG, polyacrylamide, etc., have been utilized to immobilize L-ASNase (Brumano et al., 2018). Most studies of immobilized L-ASNase have focused on its medical applications; only a few works have been devoted to the food industry. In the past few years, researchers have aimed to improve its stability to proteolysis or reduce its immunogenicity by PEGylation, fatty acid modification, and heparin-coated poly(3-hydroxybutyrate-co-3-hydroxyvalerate) nanocapsules for medical applications (Rytting, 2010; Ashrafi et al., 2013; Meneguetti et al., 2019). Moreover, covalently immobilizing L-ASNase on glutaraldehyde-activated aluminum oxide pellets could decrease the acrylamide level in potato chips by 80.5% (Agrawal et al., 2018). Another study showed that when L-ASNase-conjugated magnetic nanoparticles were incubated with 2% L-ASN and starch for 30 min, acrylamide decreased by nearly 100% in a model food system (Alam et al., 2018). Nevertheless, these two studies only focused on batch processing; continuous production processes for fluidized food components are ignored. It is necessary to design enzyme reactor models to simulate industrial applications involving the continuous catalysis of fluidized food component substrates. In addition, safety is an important factor in the food industry, and it is preferable to utilize natural nontoxic carriers and crosslinking agents to immobilize enzymes. Agarose (Aga), a natural nontoxic polysaccharide carrier with good biocompatibility, high strength, porosity, hydrophilicity, a large capacity, and easy separation, is suitable for immobilized enzyme applications (Jungbauer, 2005). N-hydroxysuccinimide (NHS) esters are an efficient and mild crosslinking agent that play a role in the displacement reaction during enzyme immobilization without being introduced itself, and they are safer than glutaraldehyde, which was used in the two previous studies (Cuatrecasas and Parikh, 1972).

A bioreactor is the key equipment needed to realize product industrialization. For immobilized enzymes, packed bed and fluidized bed reactors are mostly used. On one hand, packed bed reactors have the advantages of a high catalyst loading, high efficiency, mild flow conditions, simple structure, and easy scaling (Yizheng and Shuxiong, 2007a). On the other hand, severe flow conditions in fluidized bed reactors might damage the biocatalyst, causing a decrease in the production efficiency (Yun et al., 2010). Considering these factors and the characteristics of biocatalysts, a packed bed reactor is appropriate for substrate degradation catalysis in our experiments. Previously, L-ASNase immobilized on chitosan was used *in vitro* for continuous catalytic kinetic experiments, and a mathematical model was developed to investigate the factors of the mass transfer process in simulated blood (Hao et al., 1999; Jinin et al., 1999). However, those models mainly studied the internal and external diffusion effects of the catalyst. The entire immobilized enzyme column was not described, and the influences of the parameters of the packed bed enzyme reactor on the substrate conversion rate were not explored.

In this work, the food-grade natural polysaccharide agarose, which was activated with N-hydroxysuccinimide esters, was chosen to efficiently immobilize L-ASNase. The enzymatic properties of the immobilized enzymes were investigated, and the structure was characterized. Meanwhile, a packed bed enzyme reactor was designed, and a mathematical model was developed based on the material balance and immobilized enzyme reaction kinetics to describe the continuous catalytic process of simulated fluidized food components, which resulted in a decrease in acrylamide formation for practical use.

## Materials and Methods

### Materials

L-ASNase was purchased from Shanghai Yingxin Laboratory Equipment Co., Ltd. Four percent cross-linked agarose (Aga, 40–160 μm) was provided by Jiangsu Qianchun Biological Co., Ltd. Acrylamide (HPLC, >99%) was purchased from Sigma-Aldrich. Nessler’s reagent and L-asparagine (L-ASN) were procured from Maclin Inc. Dioxane (DIO), N,N’-dicyclohexylcarbodiimide (DCC), NHS, all other chemicals, and analytical-grade reagents were sourced from Sinopharm Chemical Reagent Co., Ltd. (Shanghai, China).

### Modification Procedure and Optimization of the Immobilization Conditions

The agarose spheres were modified with NHS according to previous work (Johansson et al., 2003). The agarose spheres were epoxidized by epichlorohydrin (ECH) in an activating solution and successively treated with ethanol and water. Then, they were carboxylated with 6-aminocaproic acid at 60°C for 2 h. The carboxylated spheres were washed with at least 10 times the volume of 30, 70, and 100% acetone and dioxane in sequence. The anhydrous carboxylated spheres were mixed with 10 ml of dioxane and then shaken at 25°C (150 r min^–1^) for 2 h.

To investigate the optimal conditions for L-asparaginase immobilization, the density and concentration of the ligand, ratio of NHS/DCC, enzyme load, and reaction time were studied. The ligand density was controlled by the ratio of ECH (v/v) in the range of 1/90 to 1/3. The enzyme load was varied from 12.8 to 64 U/ml. The NHS dosage and ratio were varied from 1 to 8% and from 1:1 to 1:2 (NHS:DCC), respectively. The immobilization time ranged from 1 to 10 h. The experiments were conducted by reacting 1 g of activated carrier with 1 ml of enzyme.

### Immobilization Procedures

**FIGURE 1:**
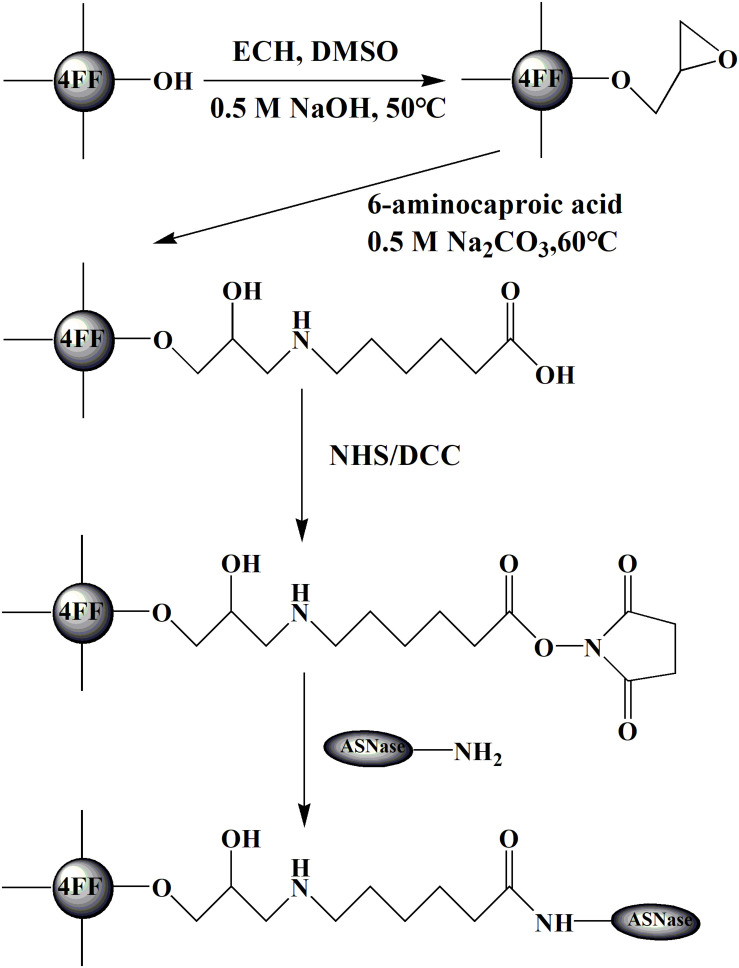
Procedures of modification and immobilization.

As shown in Figure 1, the activated agarose spheres were extensively washed with 1 mM hydrochloric acid (ice bath). The rinsed agarose spheres were mixed with a certain concentration of the enzyme solution (dissolved in 0.5 M Na_2_CO_3_ and NaCl at pH 8.0) in equal amounts (w/v) at 25°C and a speed of 150 r min^–1^ for 2 h. The nonactive groups were subsequently blocked with 0.5 M ethanolamine and extensively washed with water. Then, the immobilized enzyme was kept in 20 mM PBS (containing 0.5 M NaCl) at 4°C.

### Enzyme Activity Assay and Acrylamide Detection

The enzyme activity was assayed by estimating the ammonia by-product through nesslerization after the reaction as described earlier (Feng et al., 2017). Briefly, 300 μl of 0.15 M L-ASN was mixed with 800 μl of 20 mM PBS (pH 7.5) and incubated at 37°C for 10 min. Then, 100 μl of the free enzyme or 100 mg of immobilized ASNase (Aga-ASNase) was added to the mixture, which was incubated at 37°C for 10 min, and the reaction was stopped by adding 100 μl of 1.5 M trichloroacetic acid (TCA). Subsequently, 100 μl of the supernatant was collected by centrifugation at 8,000 rpm for 5 min and reacted with 200 μl of Nessler’s regent and 3.7 ml of distilled water in a tube. The absorbance of the solution was measured at 450 nm.

The model food system was obtained by mixing 0.1 M L-asparagine with 0.1 M starch. The effluent from the enzyme reactor was kept in a tube and heated at 180°C in an oil bath for 10 min. The samples were purified and detected using a mobile phase containing water, acetonitrile, and methanol (90:5:5, v/v/v) and a C-18 column. Acrylamide was detected by HPLC (HITACHI) using a UV diode detector.

### Characterization and Structural Analysis of Immobilized L-ASNase

The thermal stability, kinetic parameters, reusability, and storage stability of Aga-ASNase were investigated. The thermal stability was tested by preincubating the enzyme at 47 and 60°C for up to 4 h. The kinetic parameters (K_*m*_ and V_*max*_) of the free and immobilized enzymes were determined by measuring the enzyme activity at various concentrations (5–100 mM) of the substrate and using the Lineweaver–Burk plot. For the reusability, Aga-ASNase was incubated with the substrate (0.1 M L-ASN) under shaking conditions at 30°C. The supernatants were removed for enzyme activity determination. Aga-ASNase was washed three times with 10 ml of 20 mM PBS (pH 7.5). No less than six cycles were performed under the same conditions to determine the reusability.

The stabilities of the free and Aga-ASNase bioconjugates during storage at 4°C were evaluated in 20 mM PBS (pH 7.5, 0.5 M NaCl) for long times ranging from 1 to 28 days. The activity of Aga-ASNase in the first day was considered to be 100%.

The unbounded agarose spheres and Aga-ASNase bioconjugates were examined by scanning electron microscopy (SEM) at 2,400× magnification and analyzed by a Fourier infrared spectrometer. The samples were lyophilized before the experiments. The transmittance (%) was recorded in the range of 4,000 to 650 cm^–1^ with 32 scans for each sample.

### Optimization and Kinetics of the Immobilized Enzyme Reactor

The specific activity, amount of immobilized enzyme added, temperature, and velocity of flow were investigated in an adjustable-height column with an internal diameter of 1.1 cm. A certain quantity of Aga-ASNase was weighed and mixed with distilled water, the mixture was flowed into the column reactor slowly for 2 h, the liquid was then passed through at a working flow rate for 10 min, and the height of the bed was adjusted and fixed. The time was started at 0 min after a column volume flowed through. Samples (0.5 ml) were removed at the outlet at regular intervals.

To study the reaction kinetics of the enzyme reactor, a mathematical model of the enzyme reactor was developed (Xiaoyun, 2005). The Aga-ASNase spheres were columnar under the action of water compression, and the shear force could be neglected because the velocity of the flow was slow. The voidage (ε) was measured by the drainage method. Due to the high length-to-diameter ratio of the reactor, diffusion mainly occurred in the axial direction, and it was assumed that the flow and mass transfer in the reactor conformed to the one-dimensional quasi-homogeneous steady-state axial diffusion model. The apparent axial diffusion coefficient (D_*a*_) was determined by the pulse method (Shaofen et al., 2013) under the conditions of 35°C, 1 ml/min and a 0.15-m column height. In addition, the following assumptions could be made:

(1) The voidage (ε) of the packed bed reactor was assumed to be constant;
(2) The flow and mass transfer in the reactor conformed to the one-dimensional quasi-homogeneous steady-state axial diffusion model;
(3) The apparent axial diffusion coefficient D_*a*_ satisfied Fick’s law;
(4) The reaction heat was very small and could be ignored; and
(5) Substrate inhibition was neglected.

MATLAB 2018b (1994–2020 The Math Works, Inc.) was used to calculate the numerical solution of the second-order nonlinear differential equation.

## Results and Discussion

### Optimization of Immobilization Conditions

**FIGURE 2:**
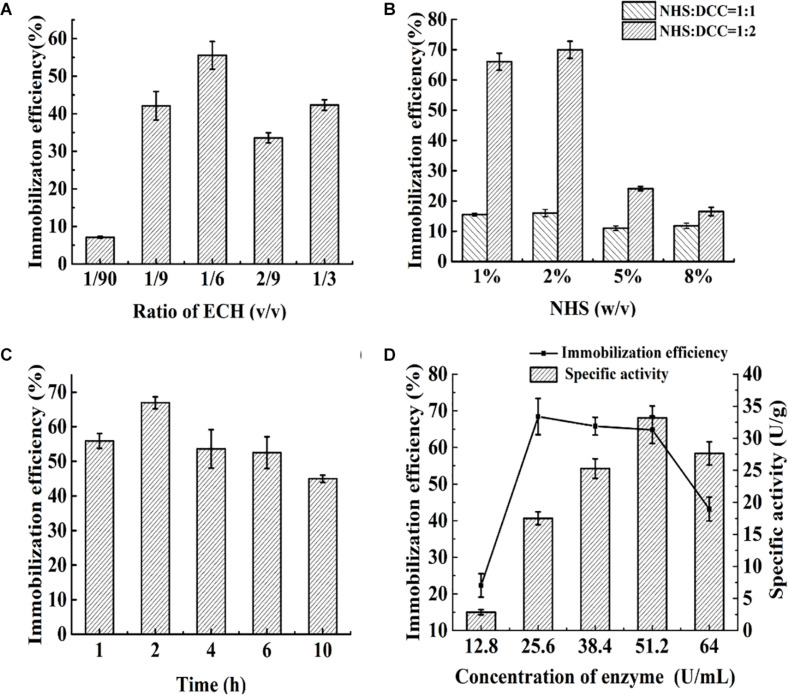
**(A)** Effect of the ratio of epichlorohydrin (ECH) (v/v) on immobilization efficiency: varied from 1/90 to 1/3. **(B)** The effect of N-hydroxysuccinimide (NHS) dosage (w/v) and ratio of NHS/N,N′-dicyclohexylcarbodiimide (DCC) (v/v) on immobilization efficiency: varied from 1 to 8% and 1:1 to 1:2, respectively. **(C)** Effect of immobilization time on immobilization efficiency: varied from 1 to 10 h. **(D)** Effect of enzyme concentration on immobilization efficiency and specific activity: varied from 12.8 to 64 U/ml.

The effects of the ligand density and concentration, ratio of NHS/DCC, enzyme concentration and reaction time on the immobilization efficiency of food-grade Aga-ASNase are presented in Figure 2. The effect of the ligand density is shown in Figure 2A; the ratio of ECH was controlled, and the peak efficiency was achieved at a ligand density of 1/6 ECH (v/v). As shown in Figure 2B, at 2% NHS (w/v), the maximum immobilization yield was obtained when the ratio of NHS:DCC was 1:2, which is different from the optimal ratio of NHS/DCC of 1:1 reported previously (Chu and Siebert, 1975). Previous studies showed that a 10- to 50-fold molar excess of the NHS ester over the amount of the amine-containing material results in sufficient conjugation (Pasta et al., 1988), and multiple NHS esters within a limited area might bind to several ε-amino groups on one enzyme, which might lead to a loss of activity to a certain degree (Meneguetti et al., 2019). Thus, in these experiments, the amount of NHS esters was controlled by the ECH ratio of 1/6 (v/v) and the NHS/DCC concentration of 2% (w/v), at which the maximum immobilization efficiency was obtained. As shown in Figures 2C,D, a maximum immobilization efficiency of 68.4% was achieved after 2 h of cross-linking. In a similar study, ASNase was immobilized on poly[N-(2-hydroxypropyl) methacrylamide] in the same way, and 47.8% of the activity was retained, whereas the estimate for PEG-ASNase was reported to be 28.9% (Monajati et al., 2019). This result indicated that ASNase immobilization with NHS esters has the advantages of rapidity, high efficiency, and mild reaction conditions. When the enzyme concentration was increased from 25.6 to 51.2 U/ml, the immobilization efficiency remained between 60 and 70%, whereas the specific activity of the catalyst nearly doubled. Based on the results in the Parameter Optimization for the Reactor and D_*a*_ Value Determination section, catalysts with a lower specific activity might be more suitable for enzyme reactors. Therefore, an enzyme concentration of 25.6 U/ml was suitable for immobilization, and the obtained catalyst was further studied.

### Characterization and Structural Analysis of the Immobilized Enzyme

**FIGURE 3:**
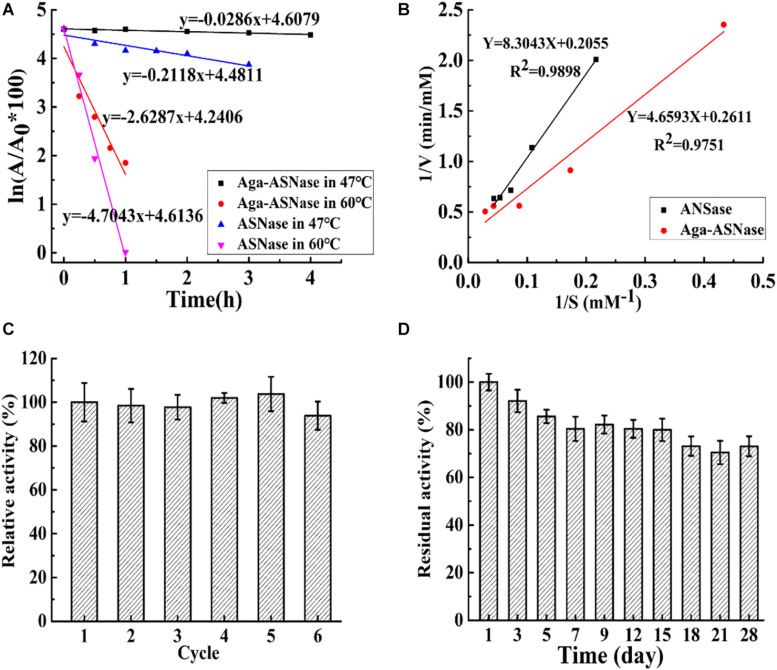
**(A)** The half-life of asparaginase (ASNase) and Aga-ASNase at 47 and 60°C, respectively. **(B)** Kinetic parameters of ASNase and agarose (Aga)-ASNase at 37°C were detected using Lineweaver–Burk plots. **(C)** The enzymatic activity assay was conducted for six reaction cycles under the conditions 30°C, pH 7.5; the carriers were recovered and washed three times with phosphoric acid buffer (pH 7.5) after each cycle, then reintroduced to the next cycles. **(D)** The catalytic activity was measured under optimal conditions (37°C, pH 7.5) for 28 days after each experimental cycle, and the catalyzer was stored at 4°C with phosphoric acid buffer (pH 7.5).

As shown in Figure 3, the thermostability, kinetic parameters, reusability, and storage stability of Aga-ASNase were investigated. In Figure 3A, the half-life of the immobilized enzyme was 7 and 1.8 times longer than that of the free enzyme at 47 and 60°C, possibly due to the greater rigidity of the enzyme conformation and the higher activation energy of unfolding at multipoint attachments for the immobilized ASNase (Wang and Cao, 2015).

The kinetic parameters of the free enzyme and Aga-ASNase were determined from the Lineweaver–Burk plots in Figure 3B. The K_*m*_ values of the free and immobilized ASNase were 40.45 and 17.6 mM, respectively. The V_*max*_ values of the free and immobilized ASNase were 4.87 and 3.83 mM min^–1^. The catalytic efficiency (V_*max*_/K_*m*_) of the immobilized enzyme was higher than that of the free enzyme. This result was largely due to the lower K_*m*_ value of the immobilized enzyme.

The reusability and storage stability of Aga-ASNase are presented in Figures 3C,D. Aga-ASNase still retained 93.21% of its initial activity after six continuous cycles, which showed its potential for application in industry. The loss of activity might be due to the processes of cleaning and filtering. Previous research showed that ASNase immobilized on magnetic particles by 5% glutaraldehyde retained 90% of its original activity after five cycles (Alam et al., 2018).

The storage period is crucial for commercial application of Aga-ASNase because it determines the viability of the immobilized enzyme over time. The free and immobilized enzymes were kept in 20 mM PBS (0.5 M NaCl) during storage. The results indicated that the free enzyme lost almost all of its activity in no more than 5 days, whereas Aga-ASNase still retained approximately 72.25% of its original activity after 28 days of storage at 4°C. Similarly, the retention of 60% of the residual activity after 30 days of storage at 25°C was previously reported (Koytepe and Ates, 2016).

**FIGURE 4:**
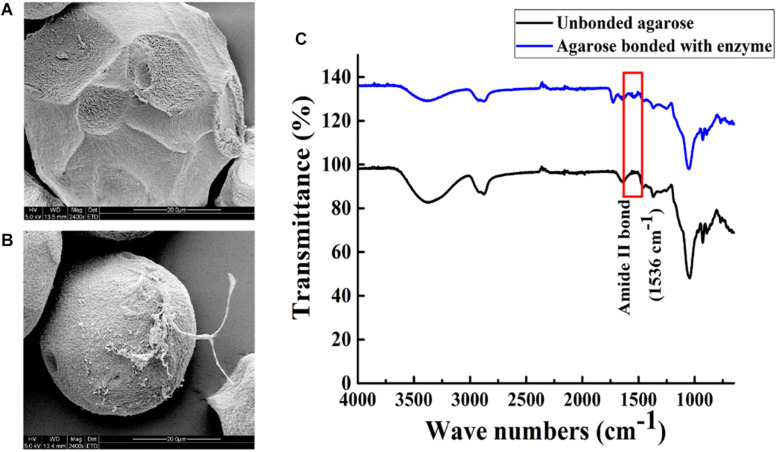
Scanning electron images (5.0 kV, 2,400×) showing the morphology image of agarose spheres before **(A)** and after **(B)** Aga-ASNase immobilization. **(C)** Fourier transform infrared (FTIR) spectra of unbonded agarose and agarose bonded with ASNase.

Agarose spheres and Aga-ASNase were scanned by SEM. Compared with the unbonded spheres shown in Figure 4A, the agarose spheres clearly exhibited surface roughness after the binding of the enzymes, as shown in Figure 4B at a high SEM magnification, and significant changes were observed. Meanwhile, the unbound spheres and Aga-ASNase conjugates were analyzed by FT-IR. Figure 4C expressly showed that the peak of the amide II bond was observed at 1,536 cm^–1^ after immobilization, confirming the binding of the ligand to the receptor by chemical bonding forces and supporting the SEM results.

### Parameter Optimization for the Reactor and D_*a*_ Value Determination

Although several reports on the degradation of acrylamide by free and immobilized L-asparaginase have appeared in the literature, few bioreactor systems have been designed for practical use. A packed bed enzyme reactor was designed, the apparent axial diffusion coefficient D_*a*_ was detected by the pulse method, and the specific activity and temperature were investigated first in preparation for later mathematical analysis of the continuous process.

**FIGURE 5:**
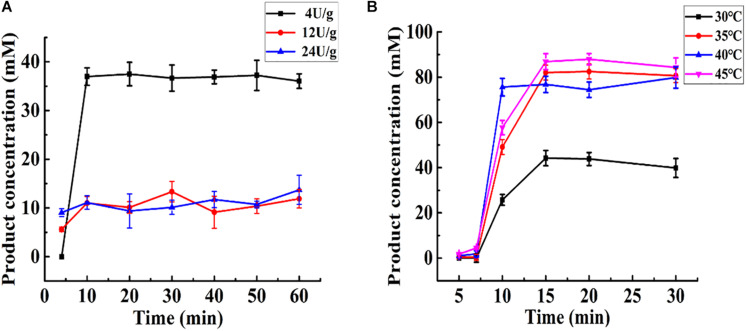
**(A)** Effect of the specific activity of Aga-ASNase on catalytic efficiency of enzyme reactor. The effluent through the reactor from 0 to 60 min was collected and detected. **(B)** The effect of temperature on catalytic efficiency of enzyme reactor. The effluent through the reactor from 0 to 30 min was collected and detected.

As shown in Figure 5A, at the same total enzyme activity (24 U), Aga-ASNase with a low specific activity (4 U/g) exhibited a higher catalytic efficiency than those with specific activities of 12 and 24 U/g, whereas the latter two exhibited nearly the same catalytic efficiency. This result might be largely due to the internal diffusion restriction (Yizheng and Shuxiong, 2007b). Moreover, the same enzyme activity (24 U) combined with a lower specific activity (4 U/g) might lead to a higher column volume, resulting in a longer reaction time. In this way, the lower the specific activity is, the higher the catalytic efficiency is at a given total enzyme activity. However, the conditions for efficient immobilization should not be ignored. Aga-ASNases with specific activities of 4, 12, and 24 U/g were obtained with immobilization efficiencies of 22.32, 68.43, and 65.81%, respectively. Thus, the optimal specific activity of Aga-ASNase was 12 U/g, and this sample was used for later research.

Changes in temperature cause an increase in the difference between the enzyme reaction rate and substrate diffusion rate. It would be somewhat complex to mathematically analyze the conversion rate as a function of temperature in a packed bed reactor. Thus, the effect of temperature was primarily determined experimentally. As shown in Figure 5B, the effect of temperature was investigated using the same amount of added immobilized enzyme. The maximum conversion rate ranged from 75.59 to 87.95% at 35, 40, and 45°C and was much higher than that at 30°C. In these experiments, 35°C was the optimal temperature for catalysis in the enzyme reactor. The later continuous analysis was conducted mathematically based on a specific activity of 12 U/g and temperature of 35°C.

**FIGURE 6:**
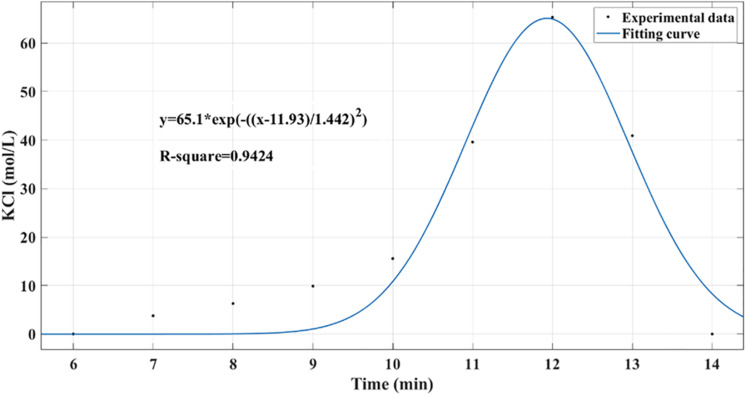
Residence time distribution curve in packed bed reactor was detected by pulse method. Potassium chloride was used as a tracer to determine the concentration distribution curve within 0 to 15 min.

As shown in Figure 6, the residence time distribution curve was detected by the pulse method (Shaofen et al., 2013), and the axial diffusion coefficient (D_*a*_) was calculated using the following formula. Here, σ_θ_^2^ is the dimensionless variance in the residence time, and Pe is the Berkeley number, which indicates the relative size of convective flow and diffusion transfer, reflecting the degree of back-mixing. The calculation result gave σ_θ_^2^ = 0.0061, Pe = 327.87, and D_*a*_ = 7.47 × 10^–8^ m^2^/s, indicating that the reactor was in a typical plug flow state under these conditions.

$$\sigma_{\theta }^{2}=\frac{2}{P\,e}-\frac{2}{P\,e^{2}}\,\left(1-e^{-P\,e}\right);\text{Pe}=\frac{u\,L}{D\,a}$$

### Kinetic Analysis and Catalytic Performance of the Immobilized Enzyme Reactor

Based on the above assumptions, a differential was taken from the bottom *h* to a column height of *H* for the mass balance calculation. The following definitions were used: *q* (ml/min) is the flow rate, *S* (mol/L) is the substrate concentration, *r* (0.55 cm) is the radius, and *p* is the conversion rate ($\text{p}=1-\frac{S}{S_{0}}$). The mathematical model was defined as follows:

$$\left(N_{i}^{F}+N_{i}^{D}\right)-\left(N_{o}^{F}+N_{o}^{D}\right)=N_{r};$$

At height *h*, the inflow of the substrate was $N_{\text{i}}^{F}=\text{q}\,S\,\varepsilon \,\pi \,{\text{r}}^{2}$, and the amount of substrate diffusion was $N_{\text{i}}^{D}=-\varepsilon \,D_{a}\,\frac{d\,S}{d\,h}\,\pi \,{\text{r}}^{2}$.

At height *h + dh*, the inflow of the substrate was $N_{\text{o}}^{F}=\text{q}\,\left(S+\text{d}\,S\right)\,\varepsilon \,\pi \,{\text{r}}^{2}$, and the amount of substrate diffusion was $N_{\text{o}}^{D}=-\varepsilon \,D_{a}\,\pi \,{\text{r}}^{2}\,\left[\frac{\text{d}\,S}{d\,h}+\left(\frac{d^{2}\,S}{d\,h^{2}}\right)\,d\,h\right]$.

The substrate reaction volume was $N_{\text{r}}=\left(1-\varepsilon \right)\,\pi \,r^{2}\,\frac{V_{m}\,S}{K_{m}+S}\,\text{dh}$.

This formula could be simplified to:

D⁢a⁢ε⁢d2⁢pd⁢h2+q⁢ε⁢d⁢pd⁢h=(1-ε)⁢Vm⁢(1-p)Km+S(1-p)0

with the boundary conditions:

$$\begin{array}{c} \text{h}=0^{-},p=0;h=0^{+},\varepsilon \,D_{\text{a}}\,\frac{d\,p}{d\,h}=q\,p;h=H,\frac{d\,p}{d\,h}=0 \end{array}.$$

**FIGURE 7:**
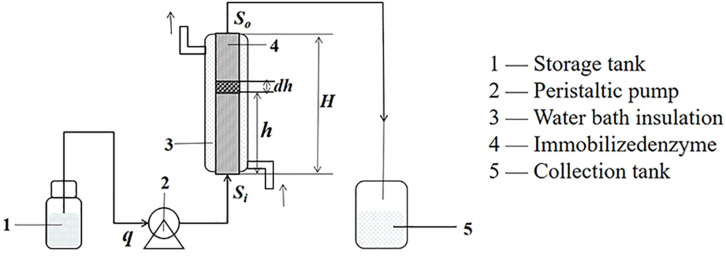
The structure diagram of an enzyme reactor.

Based on the differential equation, which showed the relationship between *p* and *q*, *h* could be obtained and verified by the experiments. The experiments were performed as shown in Figure 7. The voidage (*ε* = 0.29) of the packed bed reactor was measured by the drainage method.

**FIGURE 8:**
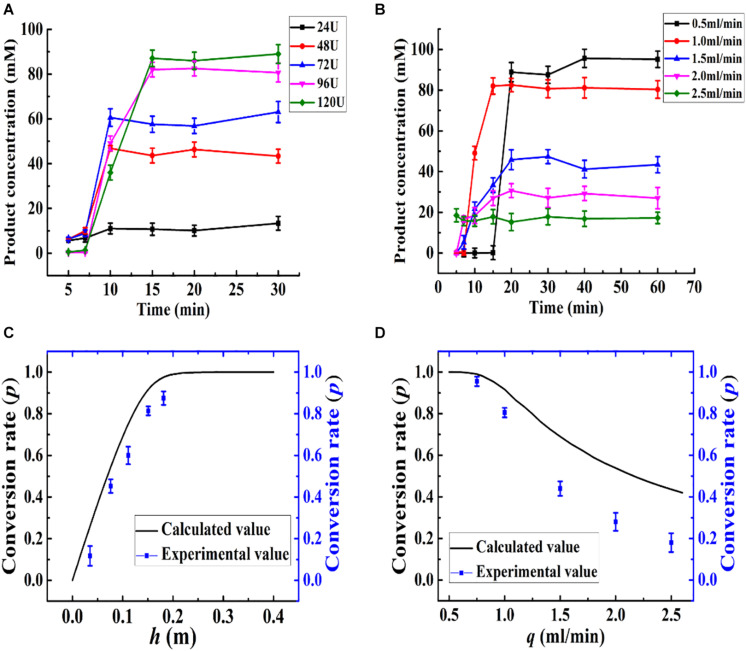
**(A)** Effect of the amount of Aga-ASNase addition on catalytic efficiency of enzyme reactor. The effluent through the reactor from 0 to 30 min was collected and detected. The Aga-ASNase addition of 24, 48, 72, 96, and 120 U was equal to the column height of 0.038, 0.075, 0.112, 0.15, and 0.185 m, respectively. **(B)** Effect of the volume flow rate on catalytic efficiency of an enzyme reactor. The effluent through the reactor from 0 to 60 min was collected and detected. **(C)** Calculated and experimental values of the effects of column height on conversion rate. **(D)** Calculated and experimental values of the effects of volume flow rate on conversion rate.

The kinetics of the immobilized enzyme reactor were determined based on the differential mass balance calculation and enzyme reaction kinetics. Based on the optimized conditions, the relationships between the calculated and experimental *p*, *h*, and *q* values are presented in Figure 8. The height of the column (*h*) was changed with the amount of Aga-ASNase added, leading to a change in the conversion rate of the effluent, as shown in Figure 8A, and the calculated value was in good agreement with the experimental value (Figure 8C). Based on both the experimental and calculated values, the maximum conversion rate changed slowly as more immobilized enzymes were added. Thus, an addition of 96 U (15-cm column height) immobilized enzymes was sufficient for the catalysis.

Then, parameter *q* was investigated, as shown in Figure 8B, and the conversion rate decreased with increasing velocity. Moreover, the experimental value appeared to be lower than the calculated value in Figure 8D when the flow rate increased more. This trend might be due to the difference between the kinetic parameters of batch and continuous reactors and the effect of the velocity on the diffusion coefficient (D_*a*_) (Zhong et al., 1993). Although the conversion rate obtained at 0.5 ml/min was somewhat higher than that at 1 ml/min, the production intensity was halved, and therefore, this flow rate was not a suitable choice. Hence, the volume flow rate was set to 1 ml/min.

According to the differential equation developed earlier, the conversion rate (*p*) is independent of the radius (*r*), and the flow rate was calculated to be a linear velocity of 1.75 × 10^–4^ m/s instead of 1 ml/min (*r* = 0.55 cm). Thus, at a velocity of no more than 1.75 × 10^–4^ m/s, the deviations between the experimental value and the calculated value could be acceptable. A high volume velocity with a linear velocity of no more than 1.75 × 10^–4^ m/s could be achieved by increasing the radius of the packed bed reactor or increasing the number of tubular reactors. In a way, the influences of the parameters (*q*, *h*) on the enzyme reactor can be described mathematically, and the mathematical model can be used for predictive purposes to decrease the number of experiments needed for catalysis optimization.

**FIGURE 9:**
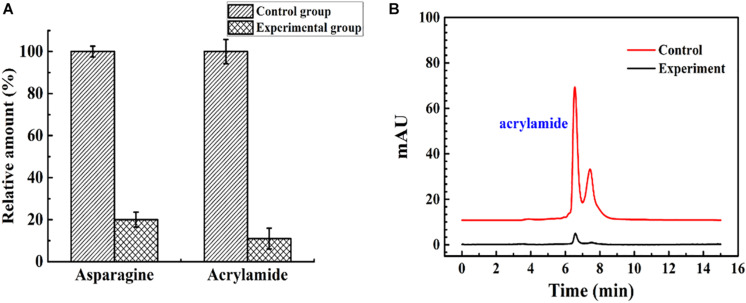
The control group and experimental group were the simulated food components that did not flow into, and flow out of, the enzyme reactor, respectively. **(A)** The relative amount of asparagine and acrylamide of the control group and experimental group. **(B)** The result of acrylamide formation in simulated system detected by HPLC.

The immobilized ASNase was used to reduce acrylamide in a model food system. The effluent flowing from the continuous catalytic process under optimal conditions (96 U, 35°C, 1 ml/min) was heated at 180°C in an oil bath for 10 min. As shown in Figure 9, compared with acrylamide formation in the untreated system, acrylamide formation was reduced by almost 89% when the fluids were flowed through the packed bed reactor. An earlier study reported that almost no acrylamide was detected after incubation with immobilized ASNase for 30 min (Alam et al., 2018). However, in this experiment, the simulated food composition passed through a 15-cm column at 1.75 × 10^–4^ m/s with an average residence time of 12 min, and an 89% decrease in acrylamide formation was obtained. To some extent, the packed bed reactor has the advantages of rapidity, high efficiency, and continuity in acrylamide reduction compared with batch processing.

## Conclusion and Discussion

In this study, L-asparaginase was immobilized on modified food-grade agarose spheres via covalent methods to decrease acrylamide in a liquid model food system. The binding of the enzyme was confirmed by SEM and FTIR, indicating that the catalytic efficiency, reusability, thermal stability, and storage stability of the immobilized L-asparaginase were significantly improved compared with those of the free enzyme. Based on the optimized temperature and specific activity, a simplified mathematical model involving the conversion rate, flow rate, and enzyme dosage was developed to assist in the design and optimization of the enzyme reactor. Moreover, simulated food components were continuously catalyzed in a packed bed reactor, resulting in an acrylamide reduction rate of nearly 89%. This work is the first to utilize food-grade agarose as the carrier and combine a mathematical model prediction with a continuous catalytic system, which enabled a rapid and efficient acrylamide control effect to be achieved.

Although the developed mathematical model was in agreement with the experimental values, large deviations were observed at high flow rates. As previously mentioned, this result might be due to differences in the kinetic parameters of the catalysts measured under batch and continuous conditions. It was speculated that when the flow rate was high, the substrate in the liquid phase failed to effectively contact the enzyme inside the carrier, resulting in nonactive enzymes in the internal space of the carrier. Using or developing a porous carrier with both a high specific surface area and high mass transfer efficiency might reduce these deviations. In this way, the enzyme reactor kinetic model might be more accurate and could be used for prediction instead of facilitating reactor optimization, which is more attractive. Furthermore, if a combination of a tubular packed bed reactor with a high conversion rate and a fluidized bed reactor with a high productivity could be designed, it might be possible to obtain greater benefits at a lower cost, and therefore, it is worthy of further study.

## Data Availability Statement

The original contributions presented in the study are included in the article/supplementary material, further inquiries can be directed to the corresponding author/s.

## Author Contributions

The experimental idea is directed by XX. The main experiment was conducted by RL with the help of XP. The manuscript was written by RL and reviewed by XX and ZZ. All authors contributed to the article and approved the submitted version.

## Conflict of Interest

The authors declare that the research was conducted in the absence of any commercial or financial relationships that could be construed as a potential conflict of interest.
